# Bioinformatics and Network Pharmacology Identify the Therapeutic Role and Potential Mechanism of Melatonin in AD and Rosacea

**DOI:** 10.3389/fimmu.2021.756550

**Published:** 2021-11-23

**Authors:** Huaxiong Zhang, Yiya Zhang, Yangfan Li, Yaling Wang, Sha Yan, San Xu, Zhili Deng, Xinling Yang, Hongfu Xie, Ji Li

**Affiliations:** ^1^ Department of Dermatology, The Second Affiliated Hospital of Xinjiang Medical University, Urumqi, China; ^2^ Department of Dermatology, Xiangya Hospital, Central South University, Changsha, China; ^3^ Department of Neurology, Second Affiliated Hospital of Xinjiang Medical University, Urumqi, China; ^4^ Hunan Key Laboratory of Aging Biology, Xiangya Hospital, Central South University, Changsha, China; ^5^ National Clinical Research Center for Geriatric Disorders, Xiangya Hospital, Central South University, Changsha, China

**Keywords:** rosacea, AD, MLT, network pharmacology, inflammation, angiogenesis

## Abstract

Rosacea is significantly associated with dementia, particularly Alzheimer’s disease (AD). However, the common underlying molecular mechanism connecting these two diseases remains limited. This study aimed to reveal the common molecular regulatory networks and identify the potential therapeutic drugs for rosacea and AD. There were 747 overlapped DEGs (ol-DEGs) that were detected in AD and rosacea, enriched in inflammation-, metabolism-, and apoptosis-related pathways. Using the TF regulatory network analysis, 37 common TFs and target genes were identified as hub genes. They were used to predict the therapeutic drugs for rosacea and AD using the DGIdb/CMap database. Among the 113 predicted drugs, melatonin (MLT) was co-associated with both RORA and IFN-γ in AD and rosacea. Subsequently, network pharmacology analysis identified 19 pharmacological targets of MLT and demonstrated that MLT could help in treating AD/rosacea partly by modulating inflammatory and vascular signaling pathways. Finally, we verified the therapeutic role and mechanism of MLT on rosacea *in vivo* and *in vitro*. We found that MLT treatment significantly improved rosacea-like skin lesion by reducing keratinocyte-mediated inflammatory cytokine secretion and repressing the migration of HUVEC cells. In conclusion, this study contributes to common pathologies shared by rosacea and AD and identified MLT as an effective treatment strategy for rosacea and AD *via* regulating inflammation and angiogenesis.

## Introduction

Rosacea is a chronic inflammatory skin disease with a global prevalence of over 5% (1). It is clinically characterized by erythema, telangiectasia, papules, or pustules and could be triggered or exacerbated by spicy food and beverages and by physical and psychological stimuli (2). Although immune and neurovascular dysregulation was reported to play an essential role in rosacea, the exact pathogenesis of rosacea remains unclear (3). Due to the lack of effective treatments, rosacea remains an incurable disease for the vast majority of patients (4).

Accumulating studies showed that rosacea develops as a manifestation of systemic illnesses that are linked to metabolic, psychiatric, and neurologic disorders, including Alzheimer’s disease (AD) (5, 6). Thyssen et al. clinically observed an increased risk of AD in patients with rosacea (6). AD is a common neurodegenerative disease, characterized by a progressive deterioration in cognitive and memory abilities, which has become a growing threat to public health (7). Studies showed that dysregulation of the inflammatory system is an essential factor to cognitive impairment in AD (8). Although chronic inflammation and vascular dysfunction are shared in the pathogenesis of both rosacea and AD (9, 10), the detailed molecular mechanism linking AD and rosacea remains limited. The common pathologies shared by rosacea and AD through molecular interaction networks may contribute to the drug discovery for rosacea treatment and AD prevention.

Melatonin (MLT), a neurohormone produced by the pineal gland, has multiple regulatory roles in circadian rhythms, sleep, and neuroendocrine activity (11, 12). In recent years, MLT has been implicated in immunomodulatory, antiangiogenic, and antioxidant activities (13, 14). MLT deficiency could increase the risk of neurodegeneration, immunoregulation disorder, and senescence (15–17). MLT levels have previously been reported to be lower in patients with AD (18, 19), which may contribute to oxidative damage of brain cells of AD patients (20, 21). Beyond that, MLT was reported as an effective treatment for AD, but the mechanisms by which MLT benefits AD are ill-defined. Interestingly, MLT is also widely applied in treating cutaneous diseases, such as atopic dermatitis (22), androgenic alopecia (23), and vitiligo (22). However, the potential therapeutic role of MLT on rosacea and its detailed pharmacological mechanisms still need to be studied.

In the present study, bioinformatics analyses revealed the biological functions, transcription factor (TF) regulatory network, and core targets in both AD and rosacea. Moreover, the network pharmacology approach identified the MLT pharmacological target network and revealed the mechanism that MLT could help in treating AD/rosacea partly by modulating inflammatory and vascular signaling pathways. Finally, the therapeutic role and mechanism of MLT were verified in rosacea.

## Materials and Methods

### Differentially Expressed Genes in AD and Rosacea

**Figure 1:**
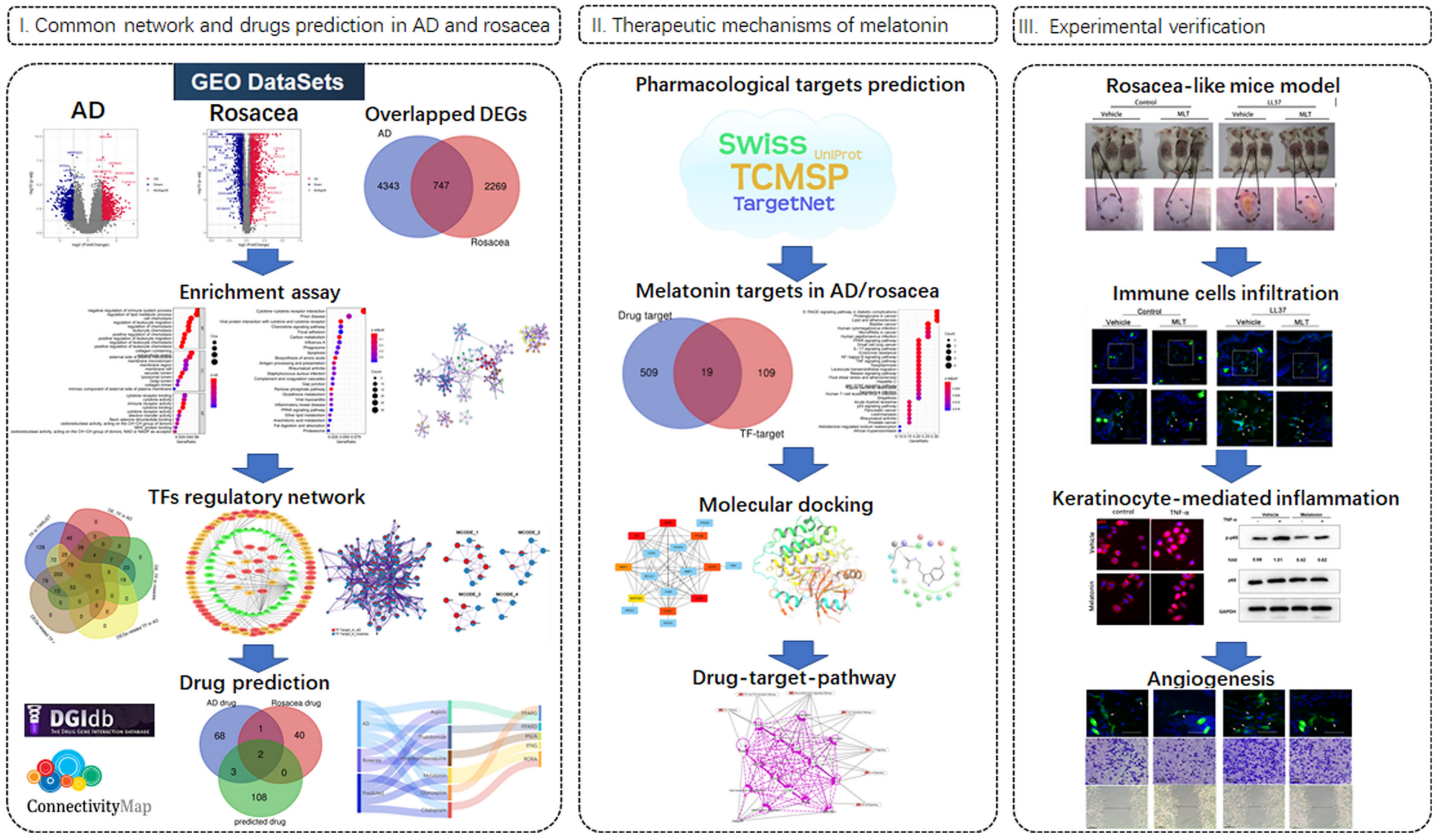
Workflow of the study.

As shown in **Figure 1**, the data were downloaded from the GEO database (https://www.ncbi.nlm.nih.gov/geo/): 23 tissues (10 hippocampus tissues with AD and 13 control hippocampus tissues) in GSE5281 (GPL570 platform) and 30 tissues (22 hippocampus tissues with AD and 8 control hippocampus tissues) in GSE28146 (GPL570 platform). The 38 rosacea tissues and 20 control tissues in GSE65914 were also downloaded from GEO. The data were standardized and the batch effects were removed using “limma” and “sva” (**Figures S1A**, **S2A**). Differentially expressed genes (DEGs) between the disease and control tissues were identified using the R “limma” package with the threshold of |logFC| >0.5 and FDR <0.05.

### Gene Ontology and Kyoto Encyclopedia of Genes and Genomes Enrichment Analyses

The Gene Ontology (GO) and Kyoto Encyclopedia of Genes and Genomes (KEGG) enrichment analyses were carried out using R “clusterProfiler”, “enrichplot”, and “ggplot2” packages.

### The Protein–Protein Interaction Networks

The protein–protein interaction (PPI) network was constructed using the STRING database (https://string-db.org/) with a combined score >0.7. “cytoHubba” was used to calculate the weight of each gene and explore the hub genes of the PPI network, and “MCODE” was used to identify the representative modules using Cytoscape software 3.8.1 (https://cytoscape.org).

### Transcription Factor–Target Networks

The TF–targets were downloaded from the TRRUST database (https://www.grnpedia.org/trrust/). The differently expressed TFs in AD and rosacea, which could regulate DEGs in AD/rosacea, were identified as key TFs. The differently expressed TFs and target genes were used to construct the TF–target networks using Cytoscape software.

### DGIbd and Connectivity Map Were Used for Drug Prediction

To prioritize the list of drugs for AD/rosacea, key TF and target genes were used for potential candidate drugs using the DGIbd database (24, 25), and then the Connectivity Map (CMap) database was also used to verify the candidate drugs for AD and rosacea (26).

### Prediction of the MLT Pharmacological Target

The potential pharmacological targets of MLT were obtained from accessible online tools, including the Traditional Chinese Medicine Systems Pharmacology Database and Analysis Platform (TCMSP), SwissTargetPrediction, and TargetNet (27). The candidate genes were corrected and identified using the UniProt database (28).

### Molecular Docking

The molecular structure of MLT (CID_4091) was downloaded from the PubChem database (https://pubchem.ncbi.nlm.nih.gov/). The protein structures of MMP9_6ESM were downloaded from the PDB database (https://www.rcsb.org/). The maestro software was used for molecular docking analysis as previously described.

### Reagents

The synthetic peptide LL37 (LLGDFFRKSKEKIGKEFKRIVQRIKDFLRNLVPRTES) used in this study was purchased from Sangon Biotech (Shanghai, China). All peptides were HPLC purified (purity >95%) and characterized by mass spectrometry. Melatonin was purchased from Sigma Company (USA). Human recombinant human TNF-α was purchased from PeproTech (USA).

### Mouse Experiment

Specific pathogen-free female BALB/c mice (6 to 7 weeks old; weight, 20–25 g) were purchased from SLAC Laboratory Animal Co., Ltd. (Shanghai, China). Before the experiment, all the animals were acclimated to experimental conditions for 1 week. The mouse model of rosacea was constructed as described before (29). All the 8-week-old BALB/c mice (7 to 8 weeks old, weight, 20–25g) were divided into four groups (control group, LL37 group, MLT group, LL37 + MLT group) randomly. Mice were sacrificed 12 h after the final injection, and mice dorsal lesion skins were collected. The inflammation severity of the rosacea mice model was evaluated according to the area of skin lesions, erythema, and thickness (30), and the skin tissues were divided into three parts for RNA extraction, HE staining, and immunofluorescence staining, respectively. Once the experiments were not to be carried out immediately, tissues were stored at −80°C until use. All mice were housed in sterile conditions at 23°C ± 2°C (12 h light/dark). All experimental protocols followed the guidelines of animal experimentation and were approved by the Ethical Committee of Xiangya Hospital of Central South University (Approval No. 201611610).

### Histological Analysis

Fresh mouse dorsal skin tissues were fixed in 4% paraformaldehyde (PFA), embedded in paraffin, and sectioned at 6 µm. Subsequently, hematoxylin and eosin (H&E) staining was performed on paraffin sections for observation under a light microscope (Olympus, Japan), and the number of dermis-infiltrating cells was then averaged and assessed statistically.

### Cell Culture and Treatment

Human immortalized keratinocyte (HaCaT) cells were cultured in complete calcium-free DMEM media (Gibco, USA) containing 10% fetal bovine serum (FBS) (Gibco, USA), 1% L-glutamine (Gibco, USA), and 1% penicillin/streptomycin (Gibco, USA), at 37°C in 5% CO_2_ in an incubator. When HaCaT cells reached 70% confluence (logarithmic phase HaCaT cells were inoculated into a 24-well plate with cell-climbing slices at the concentration of 1 × 10^4^/well, if HaCaT cells were prepared for immunofluorescence experiments), the culture medium was replaced with serum-free calcium-containing DMEM medium. After HaCaT cells were starved for 12 h, they were then treated with melatonin (1 mM) for 24 h and stimulated with 8 μM LL37 or 100 ng/ml TNF-α for 12 h, and then RNA extraction or immunofluorescence was performed. For the immunoblot experiments, after HaCaT cells reached 70% confluence, they were then treated with melatonin (1 mM) for 6 h and then stimulated with TNF-α (100 ng/ml) for 15 min, and then the cell protein was collected (the other conditions were the same as above).

Human umbilical vein endothelial (HUVEC) cells were cultured in complete RPMI 1640 medium (Thermo Fisher Scientific, USA) containing 10% FBS (Gibco, USA) and 1% penicillin/streptomycin (Gibco, USA) in an incubator (37°C, 5% CO_2_). For the detailed research methods of chemotaxis and migration assay, refer to the following specific section.

### RNA Extraction, Reverse Transcription, and RT-PCR

Total RNA in cells and tissue samples was extracted using the standard RNA extraction method with TRIzol (Invitrogen Life Technologies, USA). After a NanoDrop spectrophotometer determined RNA concentration, reverse transcription was performed on 2 μg RNA, using the Maxima H Minus First-Strand cDNA Synthesis Kit with dsDNase (Thermo Scientific, K1682, USA) to synthesize the cDNA strand, and gene expression was analyzed using an Applied Biosystems ® 7500 machine (Life Technologies, USA) with the qPCR SYBR Green Master Mix (Vazyme Biotech Co., Ltd., Nanjing, China). The primers are shown in **Table S11**.

### Immunofluorescence

Fresh mouse dorsal skin tissues were embedded in optimal cutting temperature compound and 6- to 8-μm frozen sections were cut. Then, the frozen tissues or HaCaT cells were fixed in 4% PFA for 15 min. Afterwards, the samples were permeabilized and blocked using blocking buffer (0.2% Triton-X/5% donkey serum) for 1 h. Next, the skin sections were incubated with different antibodies: rat anti-mouse CD4 antibody (1:100), rat anti-mouse CD31 antibody (1:100), and rat anti-mouse F4/80 antibody (1:100), and these antibodies were purchased from eBioscience. HaCaT cells were incubated with rabbit anti-phospho-NF-kB p65 antibody (1:200, Cell Signaling, USA) at 4°C overnight. Anti-rat Alexa 488 (1:200; Invitrogen, USA) and anti-rabbit Alexa 594 (1:200; Invitrogen, USA) were used as the secondary antibody for staining samples 1 h at room temperature. Finally, all samples were stained with 4′,6-diamidino-2-phenylindole (DAPI) for 5 min to visualize nuclei. Fluorescence images were acquired under a fluorescence microscope (Zeiss, Germany).

### Western Blot Assays

Proteins of cells were extracted with RIPA lysis buffer containing protease and phosphatase inhibitor cocktail. Then, the protein concentration was measured by BCA protein assay kits (Thermo Fisher Scientific, USA). Twenty micrograms of total protein was resolved on 10% SDS polyacrylamide gels and transferred onto a polyvinylidene fluoride (PVDF) membrane. After transferring, membranes were blocked with 5% non-fat milk and then the PVDF was incubated with the primary antibodies (rabbit anti-p65, 1:1,000; rabbit anti-phospho-p65, 1:1,000; and rabbit anti-GAPDH, 1:5,000) at 4°C overnight. After washing with TBST, the membranes were incubated with secondary horseradish peroxidase-conjugated antibodies (1:5,000, room temperature for 1 h). Finally, the immunoreactive bands were visualized using a chemiluminescent HRP substrate (Millipore, USA) and imaged using a ChemiDoc^TM^ XRS+ System (Bio-Rad, CA, USA). GAPDH expression was used as the endogenous control.

### HUVEC Cell Chemotaxis Assay

HUVEC chemotaxis was performed with transwell chambers (8 μm, Millipore, Billerica, MA, USA). In a 24-well culture plate, after being treated with 1 mM MLT or equal volumes of the vehicle solution for 24 h, HUVEC cells were seeded into each upper chamber at a density of 2 × 10^4^ cells with 100 μl serum-free RPMI 1640 medium. Then, the plate was placed in an incubator (37°C, 5% CO_2_). Non-migratory cells on the upper surface of the membrane were wiped off gently with a cotton swab after the following 24 h. Afterwards, the chamber was fixed with 4% paraformaldehyde for 15 min, and 0.1% crystal violet solution was used to stain the cells on the lower layer for 30 min. Finally, the number of invaded cells was counted under a light microscope (Olympus, Japan).

### HUVEC Cell Migration Assay

The HUVEC suspension with a 6 × 10^5^/ml density was inoculated on a six-well plate at 2 ml per well. At a confluency of 95%, the 200-μl pipetting tip was drawn vertically on the culture plate, and the non-adherent cells were rinsed with PBS three times. Then, the images were captured by using an optical microscope (Olympus, Japan) to determine the distance of scratch for 0 h. Following HUVEC incubation with RPMI 1640 medium with 3% FBS co-incubated with 1 mM melatonin or its vehicle in an incubator at 37°C for 24 h, photographs were retaken under the same conditions. The measurement tool of Image-Pro Plus software was used to measure the distance of the cell scratch boundary before and after treatment, and the distance before treatment was subtracted from the distance after treatment, which was the migration distance of the cells at 24 h.

### Statistical Analysis

All data were analyzed with SPSS 17.0 statistical software and were presented as means ± SEM. One-way ANOVA analyzed the data between groups, and the LSD method was used for pairwise comparison. *, *P* < 0.05; **, *P* < 0.01; and ***, *P* < 0.001 are considered significant.

## Results

### The DEGs and PPI network in AD and Rosacea

After integrated bioinformatics analyses for GSE5281 and GSE28146, a total of 5,091 DEGs were identified in AD tissues compared with normal tissues (**Figure S1B**). The GO analysis of DEGs was mainly related to cellular respiration, ATP metabolic process, and ATP metabolic process (**Figure S1C** and **Table S1**). Furthermore, KEGG enrichment analysis disclosed that these DEGs were significantly associated with pathways of neurodegeneration-multiple diseases, tight junction, virus infection, and so on (**Figure S1D** and **Table S2**).

Next, we constructed the PPI network and used “cytoHubba” to explore the hub genes (**Figure S2**). The top 5 representative modules were extracted from the PPI network using “MCODE”, and the module genes were enriched in mRNA splicing, clathrin-mediated endocytosis, G2/M transition, translation, G alpha (q) signaling events, and so on (**Table 1**).

**Table 1:** MCODE enrichment analysis in AD.

MCODE	GO	Description	Log10(*P*)
MCODE_1	hsa72163	mRNA splicing—major pathway	−33.9
MCODE_1	hsa72172	mRNA splicing	−33.4
MCODE_1	hsa72203	Processing of capped intron-containing pre-mRNA	−30.9
MCODE_2	hsa8856828	Clathrin-mediated endocytosis	−36.9
MCODE_2	hsa199991	Membrane trafficking	−26.4
MCODE_2	hsa5653656	Vesicle-mediated transport	−26
MCODE_3	hsa69275	G2/M transition	−33.3
MCODE_3	hsa453274	Mitotic G2-G2/M phases	−33.2
MCODE_3	hsa69278	Cell cycle, mitotic	−29.5
MCODE_4	hsa72766	Translation	−27.1
MCODE_4	ko03010	Ribosome	−22.4
MCODE_4	hsa03010	Ribosome	−22.4
MCODE_5	hsa416476	G alpha (q) signaling events	−16
MCODE_5	hsa373076	Class A/1 (rhodopsin-like receptors)	−14.5
MCODE_5	hsa500792	GPCR ligand binding	−13.3

For rosacea, 3,016 DEGs were identified in rosacea tissues compared with normal tissues (**Figure S3B**). GO enrichment indicated that rosacea was associated with immune-related biological processes (**Figure S3C** and **Table S3**). KEGG enrichment results showed immune-related pathways, the PPAR pathway, virus infection, bacterial infections, metabolism-related pathways, and so on (**Figure S3D** and **Table S4**). “cytoHubba” was used to explore the hub genes of the PPI network (**Figure S4**), and the top 5 representative modules were extracted from the PPI network using “MCODE” (**Figure S4**). The module genes were enriched in G alpha (i) signaling events, interferon signaling, neutrophil degranulation, cornification, neutrophil degranulation, and so on (**Table 2**).

**Table 2:** MCODE enrichment analysis in rosacea.

MCODE	GO	Description	Log10(*P*)
MCODE_1	hsa418594	G alpha (i) signaling events	−55
MCODE_1	hsa373076	Class A/1 (rhodopsin-like receptors)	−51.0
MCODE_1	hsa375276	Peptide ligand-binding receptors	−50.8
MCODE_2	hsa913531	Interferon signaling	−42.1
MCODE_2	hsa1280215	Cytokine signaling in the immune system	−30.7
MCODE_2	GO:0034341	Response to interferon-gamma	−25.3
MCODE_3	hsa6798695	Neutrophil degranulation	−32.4
MCODE_3	GO:0043312	Neutrophil degranulation	−32.3
MCODE_3	GO:0002283	Neutrophil activation involved in immune response	−32.2
MCODE_4	GO:0070268	Cornification	−43.8
MCODE_4	hsa6809371	Formation of the cornified envelope	−42.6
MCODE_4	hsa6805567	Keratinization	−38.5
MCODE_5	hsa6798695	Neutrophil degranulation	−12.4
MCODE_5	GO:0043312	Neutrophil degranulation	−12.4
MCODE_5	GO:0002283	Neutrophil activation involved in immune response	−12.3

### The Co-Expressed DEGs and Enriched Pathways in Both AD and Rosacea

**Figure 2:**
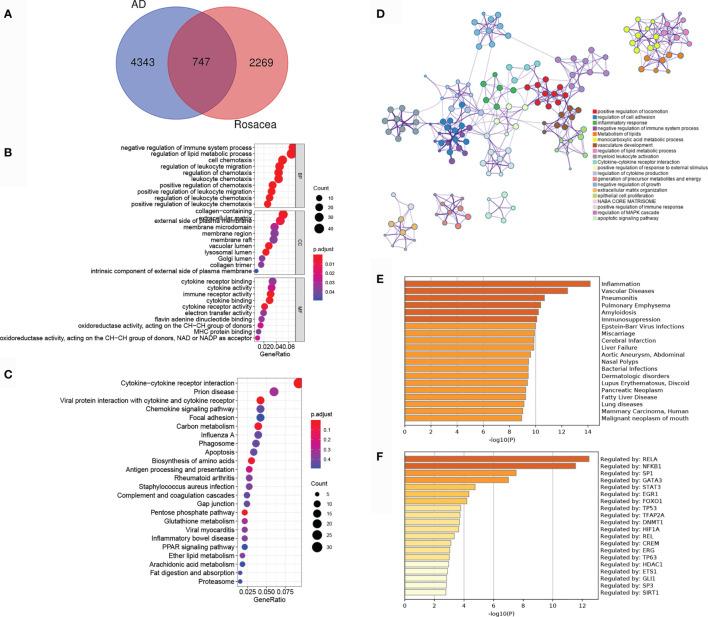
The co-differentially expressed genes (DEGs) in Alzheimer’s disease (AD) and rosacea. **(A)** The Venn graph of ol-DEGs in both AD and rosacea. **(B)** GO enrichment analysis of ol-DEGs. **(C)** The KEGG analysis of co-DEGs. **(D)** The pathway enrichment analysis of co-DEGs using Metascape. **(E)** The disease enrichment analysis of ol-DEGs in DisGeNET using Metascape. **(F)** The transcription factor (TF) enrichment analysis of ol-DEGs in TRRUST using Metascape.

To reveal the shared regulatory network, 747 overlapped DEGs (ol-DEGs) were detected in AD and rosacea (**Figure 2A**). GO enrichment analysis showed that the ol-DEGs were related to immune-related processes and lipid metabolic processes (**Figure 2B**). The KEGG enrichment results showed that ol-DEGs were related to cytokine/chemokine pathways, infectious/inflammatory disease, and metabolism-related pathways (**Figure 2C**). Consistent with these results, inflammation-, metabolism-, and apoptosis-related pathways were enriched using the Metascape database (**Figure 2D**). Moreover, the enrichment analysis showed that ol-DEGs were related to inflammation-, vascular-, and infection-related diseases in DisGeNET (**Figure 2E**). Transcription factor–target analysis showed that many ol-DEGs were regulated by TFs in the TRRUST database (**Figure 2F**), indicating that the TF regulatory network may play an essential role in the progression of AD and rosacea.

### The TF Regulatory Network and Potential Drugs for AD and Rosacea

**Figure 3:**
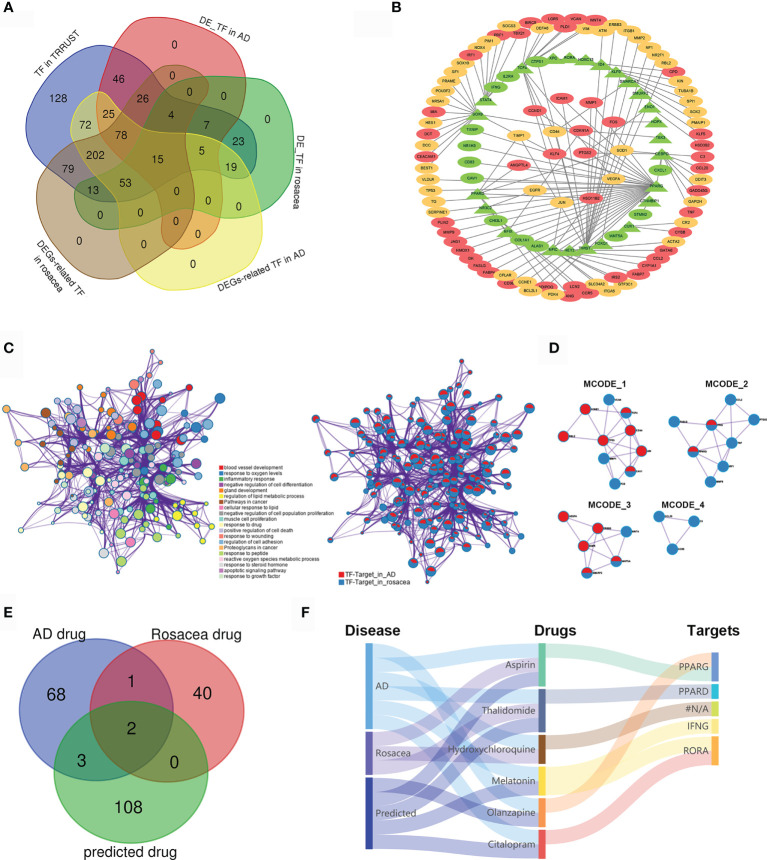
The TF regulatory network and candidate drugs for AD and rosacea. **(A)** The common TFs in AD and rosacea. **(B)** The TF regulatory network in AD and rosacea. **(C)** The enrichment of TF–target genes using the Metascape database. Network of enriched terms colored by cluster ID and the identities of the gene lists. **(D)** MCODE components identified in TF–targets in rosacea and AD. **(E)** The Venn diagram revealed the intersection among AD drugs, rosacea drugs, and predicted drugs. **(F)** The Sankey diagram revealed the correlation between the disease, drugs, and targets.

To further reveal the TF regulatory network in both AD and rosacea, we analyzed the differently expressed TFs with potential targets differently expressed in AD and rosacea. Twenty-four hub TFs are identified in **Figure 3A**. The TF regulatory network in AD and rosacea is shown in **Figure 3B** and **Table S5**. The TFs and targets were enriched in inflammatory-, blood vessel development-, and apoptosis-related signaling pathways (**Figure 3C**). In **Figure 3D**, four models were observed to be enriched in atherosclerosis-, IL-17-, proteoglycan-, and inflammatory response-related signaling pathways (**Figure 3D** and **Table 3**). These results indicated that inflammatory- and blood vessel-related pathways are crucial during the pathogenesis of AD and rosacea.

**Table 3:** MCODE enrichment analysis of TF–targets in rosacea and AD.

MCODE	GO	Description	Log10(*P*)
MCODE_1	hsa05200	Pathways in cancer	−6.9
MCODE_1	ko05418	Fluid shear stress and atherosclerosis	−6.9
MCODE_1	hsa05418	Fluid shear stress and atherosclerosis	−6.8
MCODE_2	ko04657	IL-17 signaling pathway	−10.7
MCODE_2	hsa04657	IL-17 signaling pathway	−10.7
MCODE_2	hsa04668	TNF signaling pathway	−10.2
MCODE_3	ko05205	Proteoglycans in cancer	−10.0
MCODE_3	hsa05205	Proteoglycans in cancer	−9.8
MCODE_3	GO:2000243	Positive regulation of reproductive process	−9.0
MCODE_4	GO:0009617	Response to bacterium	−4.8
MCODE_4	GO:0006954	Inflammatory response	−4.7

Next, we analyzed the potential drugs for AD and rosacea. Using the DGIbd database (https://dgidb.org/), 12 of the 37 hub genes were observed as targets of the 113 predicted drugs (**Table S6**). We compared the 74 drugs for AD, the 44 drugs for rosacea, and the 113 predicted drugs for hub genes. Three drugs (aspirin, thalidomide, and hydroxychloroquine) overlapped in AD and rosacea, and two (aspirin and thalidomide) of these were observed in the predicted drugs (**Figure 3E**), proving that the candidate drugs for AD/rosacea have high credibility. Moreover, three predicted drugs (MLT, olanzapine, and citalopram) have been used for AD treatment. The Sankey diagram revealed the correlation between disease, drugs, and targets (**Figure 3F**). These results indicated that the three predicted drugs (MLT, olanzapine, and citalopram) could be effective therapeutic strategies for rosacea. Among these potential drugs, MLT was co-associated both with retinoid-related orphan receptor alpha (RORA) as well as IFN-γ. MLT was predicted as a candidate drug using the CMap database (**Table S7**). So, MLT was selected for further function assay.

### Identifying MLT Targets in AD and Rosacea

**Figure 4:**
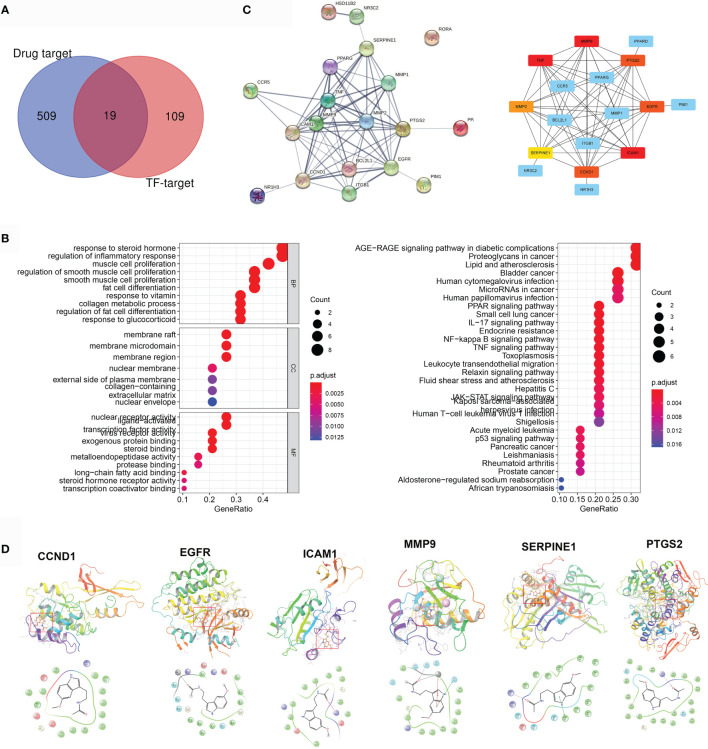
Pharmacological targets of melatonin (MLT) in AD and rosacea. **(A)** Nineteen intersection genes of MLT against AD/rosacea. **(B)** GO and KEGG enrichment analysis of the 19 MLT targets. **(C)** PPI network of the 19 MLT targets. **(D)** Molecular docking revealed the binding of MLT to its targets.

Five hundred twenty-nine pharmacological targets of MLT were predicted using the TCMSP, TargetNet, and SwissTargetPrediction databases, and then the repetitive genes were deleted using the UniProt database. An overlap of 128 AD/rosacea TF–targets with MLT pharmacological targets identified 19 intersection genes of MLT against AD/rosacea (**Figure 4A** and **Table S8**). GO analyses of the 19 genes indicated that MLT could affect the regulation of inflammatory response, collagen metabolic process, response to oxygen levels, vascular-associated smooth muscle cell proliferation, and so on (**Figure 4B** and **Table S9**). Additionally, 51 KEGG pathways were significantly enriched (*P*-adjusted < 0.05), including the AGE-RAGE signaling pathway in diabetic complications, lipid metabolism and atherosclerosis, NF-kappa B signaling pathway, IL-17 signaling pathway, PPAR signaling pathway, and TNF signaling pathway (**Figure 4B** and **Table S10**). Next, the PPI network of the 19 intersection targets was analyzed using STRING (**Figure 4C**), cytoHubba was used to identify eight core gene targets, namely, *CCND1*, *EGFR*, *ICAM1*, *MMP2*, *MMP9*, *PTGS2*, *SERPINE1*, and *TNF* (**Figure 4C**).

Next, molecular docking was performed to identify the possible binding between MLT and eight core targets. The result showed that MLT could bind to *CCND1*, *EGFR*, *ICAM1*, *MMP9*, *PTGS2*, and *SERPINE1* with docking scores of −5.86, −7.16, −6.02, −5.95, −6.52, and −6.81, respectively (**Figure 4D**).

## The Potential MLT Alleviated Rosacea-Like Phenotypes in Mice

**Figure 5:**
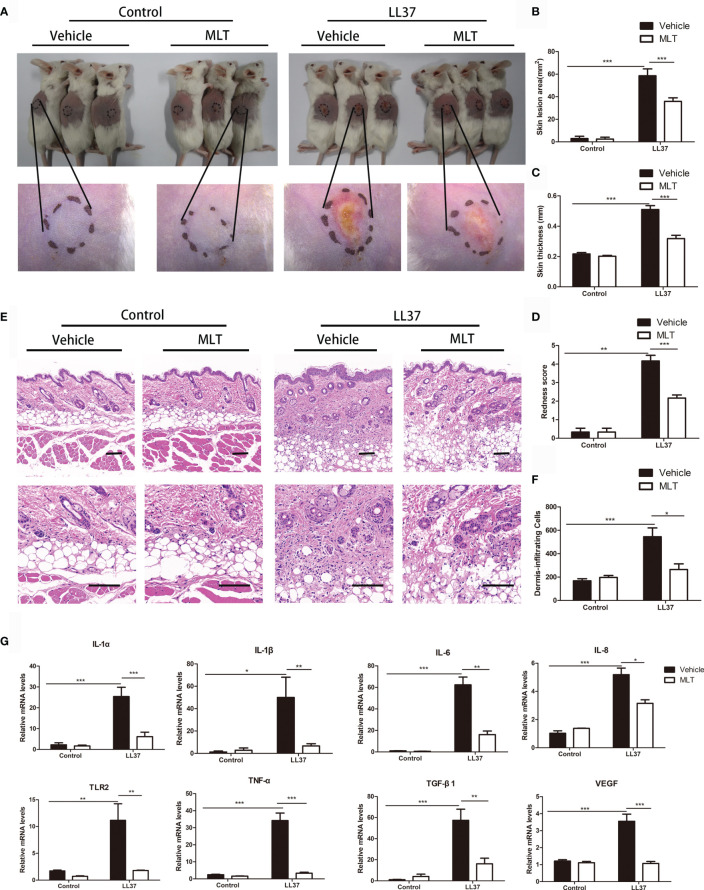
The treatment effects of MLT on rosacea. **(A)** MLT significantly alleviated rosacea-like phenotype in mice. The effects of MLT on lesion area **(B)**, skin thickness **(C)**, and redness score **(D)** in rosacea-like mice. **(E)** H&E staining of rosacea-like lesion. Scale bars: 100 μm. **(F)** Dermal inflammatory cell infiltration was quantified in rosacea-like mice. **(G)** The mRNA expression of rosacea-related markers in mice. All results are representative of at least three independent experiments. Data expressed as individual values with mean ± SEM. *P < 0.05, **P < 0.01, ***P < 0.01.

To verify the therapeutic effect of MLT on rosacea, rosacea-like mice were treated with MLT for 4 days (29). We found that MLT significantly ameliorated LL37-induced rosacea-like phenotypes (**Figure 5A**). The redness area, redness score, skin thickness, and inflammatory cell infiltration were dramatically decreased by MLT treatment (**Figures 5B–F**). Moreover, MLT repressed the expression of proinflammatory cytokines, such as IL-6, TNF-α, TLR2, TGF-β1, MMP9, and VEGF in rosacea-like dermatitis (**Figure 5G**).

**Figure 6:**
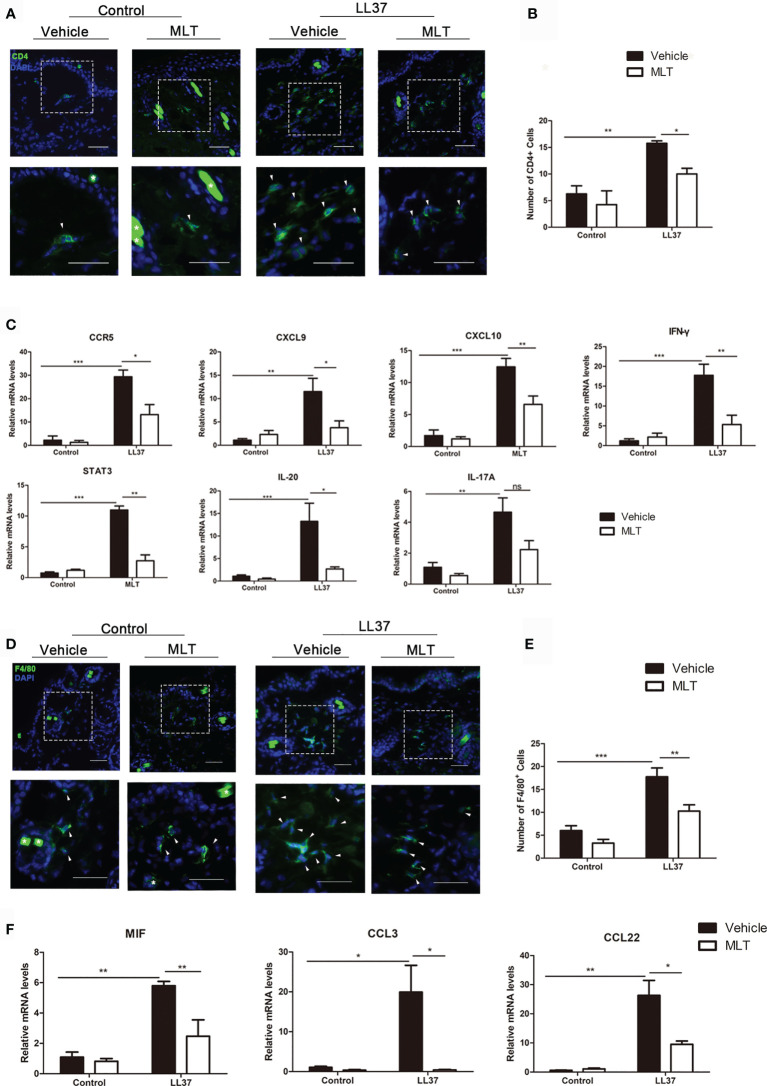
MLT inhibits immune cell infiltration in rosacea-like mice. **(A)** Immunostaining of CD4^+^ T cells in rosacea-like mice. Scale bar: 50 μm. **(B)** The infiltrated CD4^+^ T cells were quantified. **(C)** qPCR analysis detected the expression of Th1- and Th17-related genes in rosacea-skin lesions. **(D)** Immunostaining of macrophages in rosacea-like mice. Scale bar: 50 μm. **(E)** The infiltration of macrophage cells was quantified in rosacea-like mice. **(F)** qPCR analysis detected the expression of macrophage-related genes. All results are representative of at least three independent experiments. Data expressed as individual values with mean ± SEM. *P < 0.05, **P < 0.01, ***P < 0.01.

As described in previous studies, the infiltration of CD4^+^ T cells and Th1/Th17 polarized cells is essential in the pathogenesis of rosacea (31–33). In this study, immunofluorescence and qPCR analysis showed that MLT significantly reduced the infiltration of CD4^+^ T cells in rosacea-like dermatitis (**Figures 6A, B**) and suppressed the expression of Th1-related genes (*IFN-γ*, *CCR5*, *CXCL9*, *CXCL10*) and Th17-related genes (*STAT3* and *IL-20*) (34) in rosacea-like dermatitis (**Figure 6C**).

Macrophages have also played an important role in rosacea (35). To investigate whether MLT can inhibit macrophage activity in rosacea-like dermatitis, we detected the infiltration of F4/80^+^ cells. The number of infiltrated F4/80^+^ cells was significantly attenuated (**Figures 6D, E**) and the expression of macrophage-related genes (*MIF*, *CCL3*, *CCL22*, and *CCR4*) was evidently reduced by MLT treatment in rosacea-like dermatitis (**Figure 6F**). Collectively, these data suggested that MLT suppressed the immune response in rosacea.

### MLT Reduced the Secretion of Inflammatory Factors From Keratinocytes

**Figure 7:**
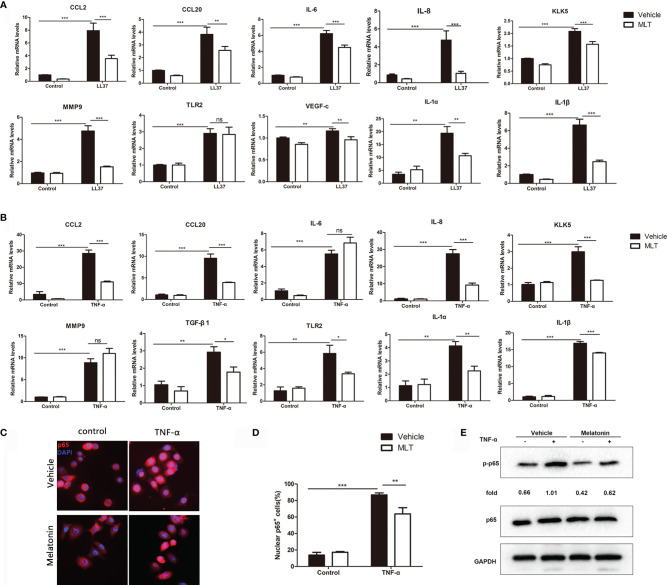
MLT reduced the secretion of inflammatory factors from keratinocyte. qPCR analysis revealed the effects of MLT on the expression of inflammatory cytokines in LL37-treated HaCaT cells **(A)** and TNF-α-treated HaCaT cells **(B)**. **(C)** Immunofluorescence analysis revealed the TNF-α-induced p65 translocation. **(D)** Percentage of p65-positive cells in the nucleus. **(E)** Immunoblotting of p-p65 and p65 HaCaT cells. All results are representative of at least t independent experiments. Data expressed as individual values with mean ± SEM. *P < 0.05, **P < 0.01, ***P < 0.001. A two-tailed unpaired Student’s *t*-test was used.

Studies showed that keratinocyte-mediated secretion of inflammatory chemokines and cytokines plays a pivotal role in the progression of rosacea (29). Here, we explored the effects of MLT on chemokine and cytokine expression in LL37-treated HaCaT cells. The results showed that 1 mM MLT significantly reduced LL37-induced CCL2, CCL20, MMP9, KLK5, IL-6, IL-8 and VEGF-c expression (**Figure 7A**). What is more, the expression of NF-κB downstream genes (IL-1α and IL-1β) was significantly repressed by MLT. To further assess the precise anti-inflammatory mechanism of MLT, we stimulated the HaCaT cells with TNF-α (100 ng/ml), one of the most potent inducers of NF-κB activation (36). We found that TNF-α induced the mRNA levels of chemokines and cytokines, including CCL2, CCL20, IL-8, KLK5, TGF-β1, TLR2, IL-1α, and IL-1β, which were abrogated by MLT treatment obviously (**Figure 7B**). Moreover, immunofluorescence results also revealed the inhibition of MLT on TNF-α-induced p65 translocation (**Figures 7C, D**) and phosphorylation of p65/NF-κB (**Figure 7E**). Collectively, these data suggest that MLT ameliorated HaCaT-mediated inflammation partly by modulating NF-κB signaling.

### MLT Decreases Angiogenesis by Repressing the Migration and Chemotaxis of HUVEC

**Figure 8:**
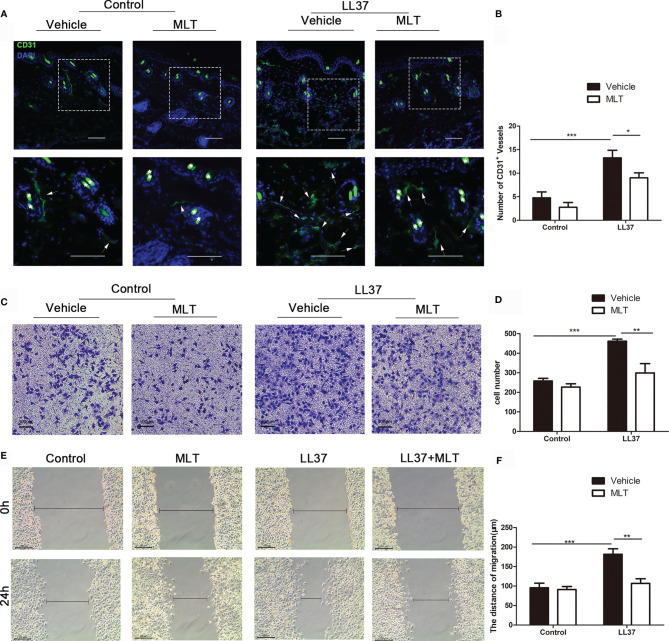
MLT suppresses angiogenesis in rosacea. **(A)** Immunostaining of CD31^+^ cells in rosacea-like lesions. Scale bar: 50 μm. **(B)** The CD31^+^ microvessels were quantified. **(C, D)** The transwell assay was used to detect the chemotaxis ability of HUVEC cells. **(E, F)** The transwell assay was used to detect the migration ability of HUVEC cells. All results are representative of at least three independent experiments. Data expressed as individual values with mean ± SEM. *P < 0.05, **P < 0.01, ***P < 0.01. One-way ANOVA with Bonferroni’s *post-hoc* test or two-tailed unpaired Student’s *t*-test was used.

We next analyzed the effects of MLT on angiogenesis in rosacea using immunostaining. As shown in **Figures 8A, B**, the number of CD31^+^ vessels was significantly reduced by MLT treatment. MLT suppressed LL37-induced HUVEC chemotaxis (**Figures 8C, D**) and migration (**Figures 8E, F**). Collectively, these data provide novel evidence that MLT may be a promising therapeutic strategy for vascular dysfunction in rosacea.

## Discussion

Rosacea is a chronic facial inflammatory skin disease, with a global prevalence of over 5% (1). Due to the lack of effective and safe treatments, rosacea may develop as a manifestation of systemic diseases and is significantly associated with metabolic, psychiatric, and neurologic disorders, including AD (37). Therefore, a novel, effective, and safe therapeutic strategy for rosacea is urgently needed. This study revealed the common TF regulatory network in AD/rosacea and identified MLT as a candidate drug for rosacea. The therapeutic effect and mechanism of MLT were verified *in vivo* and *in vitro*, and the network pharmacology analysis revealed the MLT-related molecular functions and pharmacological targets for treating AD and rosacea.

Although the exact etiology of rosacea is still uncertain, it is well known that vascular and immunologic dysregulation play a crucial role in rosacea pathogenesis. Chronic neuroinflammation is essential for the pathogenesis of AD significantly (6). Egeberg et al. observed an increased risk of AD in patients with rosacea, partly because of the overlap of proinflammatory mediators between rosacea and AD (6). Consistent with rosacea, vascular dysfunction was also observed in AD (9). In this study, 747 ol-DEGs were detected in AD and rosacea, which were enriched in immune- and metabolism-related pathways. Moreover, the enrichment analysis showed that ol-DEGs were related to inflammation-, vascular-, and infection-related diseases in DisGeNET. These results indicated that inflammatory- and blood vessel-related pathways play a critical role in the pathogenesis of AD and rosacea.

Based on the ol-DEGs, we revealed the TF regulatory network in AD/rosacea, indicating a central role of the 24 TFs in regulating the pathogenesis of AD and rosacea, including PPARG, STAT4, sox9, and RORA. PPARG (PPARγ), a member of the nuclear receptor superfamily, is mainly expressed in adipose tissues and regulates lipid metabolism (38). Recently, studies described the regulation of PPARγ on inflammation by inhibiting cytokines and MMPs and regulating oxidative stress-sensitive pathways and NF-κB pathways (38). Moreover, PPARγ plays a crucial role in the pathogenesis of AD by regulating mitochondrial function, and it is also considered a promising target for pharmacological-based therapies (39). STAT4 is an essential mediator of inflammation by regulating IFN-γ (40, 41), which is closely related to the progression of AD and rosacea (32, 42). RORA, a lipid-sensing nuclear receptor, was reported to have diverse biologic functions, including the regulation of inflammation, lipid metabolism, and angiogenesis (43–46). RORA is distinctly upregulated and reported to play a central role in AD (47). Taken together, these key TFs could be involved in the regulation of inflammation and angiogenesis by targeting their target genes in AD and rosacea. Then, the key TFs and their targets were subsequently submitted to the DGIdb database for predicting the drugs for AD and rosacea, and 113 candidate drugs were identified. In AD and rosacea, two (aspirin and thalidomide) of the three overlapped drugs (aspirin, thalidomide, and hydroxychloroquine) were observed in the predicted drugs, proving that the candidate drugs for AD/rosacea have high credibility. Moreover, three predicted drugs (MLT, olanzapine, and citalopram) have been used for AD treatment. Among these, MLT was found to be the drug that could target both RORA as well as IFN-γ. Moreover, the potential therapeutic effect of MLT on AD and rosacea was also verified using CMap. These findings indicated that MLT could be an effective therapeutic strategy for rosacea.

MLT is a neurohormone that acts as the major regulator of the daily biological rhythm (48) and plays an essential role in diverse physiological processes, including the aging process (13), neuroprotection (49), immune regulation (50), and repressing angiogenesis (51). MLT is safe, has low toxicity, and shows beneficial action against various diseases including AD (52, 53). Hossain et al. showed that MLT can improve sleep quality to mitigate AD neuropathology (54). Our previous study showed that rosacea patients presented poorer sleep quality, which might subsequently aggravate rosacea through regulating inflammatory factors (55). So, we speculate that MLT may relieve rosacea partly by regulating sleep quality.

MLT normally orchestrates daily and seasonal rhythms by acting on the MT(1) and MT(2) receptors (56). Moreover, RORα and RORβ, nuclear MLT receptors, are involved in MLT-mediated regulation of pathological and physiological cardiac hypertrophy (57). Recently, several direct MLT targets and interacting proteins were identified in AD and SARS-CoV-2 treatment, including DAPK1, calmodulin (CALM) 1, and CALM 2 (58, 59). To further reveal the potential molecular mechanism of MLT on AD/rosacea treatment, we used network pharmacology methods to analyze the detailed anti-AD/anti-rosacea mechanisms of MLT. Nineteen genes in the TF regulatory network were identified as potential pharmacological targets of MLT against AD and rosacea. The GO/KEGG enrichment results indicated that anti-AD and anti-rosacea effects exerted by MLT were directed *via* regulating vascular-associated signaling pathways and inflammation-related signaling pathways, including IL-17, NF-κB, and TNF. Based on the above results, we detected the therapeutic role of MLT on rosacea. The results showed that MLT significantly improved the rosacea-like phenotype *in vivo*. MLT treatment reduced the CD4^+^ T-cell and macrophage infiltration and Th1/Th17 polarization partly by repressing keratinocyte-mediated cytokine secretion. Moreover, MLT dramatically suppressed the angiogenesis in the rosacea-like mouse model, partly by inhibiting chemotaxis and migration of HUVECs and VEGF expression. The NF-κB signaling pathway was reported to be activated and functioned as a therapeutic target in AD and rosacea (60–62). MLT was reported to repress the NF-κB signaling pathway in treating neuroinflammation and neurodegeneration (63). In this study, we found that MLT inhibited the activation of the NF-κB pathway in TNF-α-induced inflammatory HaCaT cell, and MLT remarkably decreased the expression of NF-κB downstream genes, *IL-1α* and *IL-1β*, in LL37- or TNF-α-treated HaCaT cells. Moreover, TNF-α and IL-17A were also reported as the key cytokines in AD and rosacea (32, 64, 65). Here, we also noted the repression of MLT on IL-17A/TNF-α in rosacea-like mice. These results indicated that MLT attenuates the inflammation of AD/rosacea partly *via* the NF-κB/IL-17 pathways. Considering the repression of MLT on IL-17, NF-κB, and TNF signaling pathways, MLT could be a potential candidate treatment for patients with autoimmune diseases including psoriasis and vitiligo.

Finally, molecular docking analysis revealed the direct MLT targets, including *MMP9*, *CCND1*, *EGFR*, *ICAM1*, *PTGS2*, and *SERPINE1*, which were inflammation- and angiogenesis-related genes. *MMP9* was reported as a key inflammatory factor and as a therapeutic target in both rosacea and AD. Notably, some drugs with an MMP inhibitor function, such as doxycycline and tetracycline, were used for rosacea treatment (66), and the *MMP9* inhibitor improved specific neurobehavioral deficits in AD mouse (67). CyclinD1 (*CCND1*), a prime amplified gene in various cancers (68), was also reported to participate in angiogenesis by repressing the proliferation of endothelial cells (69). *ICAM1*, an intercellular adhesion molecule, exerts proinflammatory actions *via* facilitating adhesion and subsequent transmigration of leukocyte (70), increasing the permeability of endothelial and epithelial barrier (71), and promoting immune cell activity (72). The expression of *ICAM1* was upregulated in rosacea and AD (73, 74). *SERPINE1*, a serpin peptidase inhibitor, is related to immune response in gastric cancer (75). *PTGS2*, also known as COX-2, has a critical role in initiating inflammatory response and angiogenesis (76, 77). Moreover, studies demonstrated the potential relationship between MLT target genes (*MMP9*, *CCND1*, *EGFR*, *ICAM1*, *PTGS2*, and *SERPINE1*) and rosacea-related key genes (*IL-6*, *IL-1β*, *IFNγ*, *IL-17*, and *TNF-α*) (78, 79). So, we speculated that MLT repressed the IL-17, NF-κB, and TNF signaling pathways by targeting these pharmacological targets, subsequently leading to inflammation and angiogenesis in AD and rosacea (**Figure S5**).

## Conclusions

In this study, bioinformatics analysis revealed the shared TF regulatory network and the potential drugs for AD and rosacea. Moreover, the potent pharmacological targets and the therapeutic mechanism of MLT against AD/rosacea were identified by network pharmacology and verified in *in vivo*/*in vitro* experiments. In conclusion, this study contributes to the common pathologies shared by rosacea and AD and identified MLT as an effective treatment strategy for rosacea and AD *via* regulating inflammation and angiogenesis.

## Data Availability Statement

The original contributions presented in the study are included in the article/**Supplementary Material**. Further inquiries can be directed to the corresponding authors.

## Ethics Statement

All experimental protocols followed the guidelines of animal experimentation and were approved by the Ethical Committee of Xiangya Hospital of Central South University (Approval No. 201611610).

## Author Contributions

YZ, HZ, and JL conceived and designed the study. YZ, YL, YW, SY, and SX performed the data analysis and data interpretation. YZ, ZD, and XY conducted the bioinformatics and statistical analyses. YZ, HZ, and JL prepared the manuscript. YZ and HZ performed the cell experiments and animal experiments and data analysis. All authors contributed to the article and approved the submitted version.

## Funding

This work was supported by the National Natural Sciences Foundation of Hunan Province (2020JJ5950), the National Natural Science Foundation of China (81703149 and 82073457), and the Science and Technology Innovation Plan of Hunan Province (No. 2018SK2087).

## Conflict of Interest

The authors declare that the research was conducted in the absence of any commercial or financial relationships that could be construed as a potential conflict of interest.

## Publisher’s Note

All claims expressed in this article are solely those of the authors and do not necessarily represent those of their affiliated organizations, or those of the publisher, the editors and the reviewers. Any product that may be evaluated in this article, or claim that may be made by its manufacturer, is not guaranteed or endorsed by the publisher.
