# Supplementary material for: Bioinformatics and Network Pharmacology Identify the Therapeutic Role and Potential Mechanism of Melatonin in AD and Rosacea
Source: Front Immunol. 2021 Nov 23;12:756550. doi: 10.3389/fimmu.2021.756550 (PMC8657413; doi:10.3389/fimmu.2021.756550)
Supplement: Supplementary file 1 [file DataSheet_1.docx]

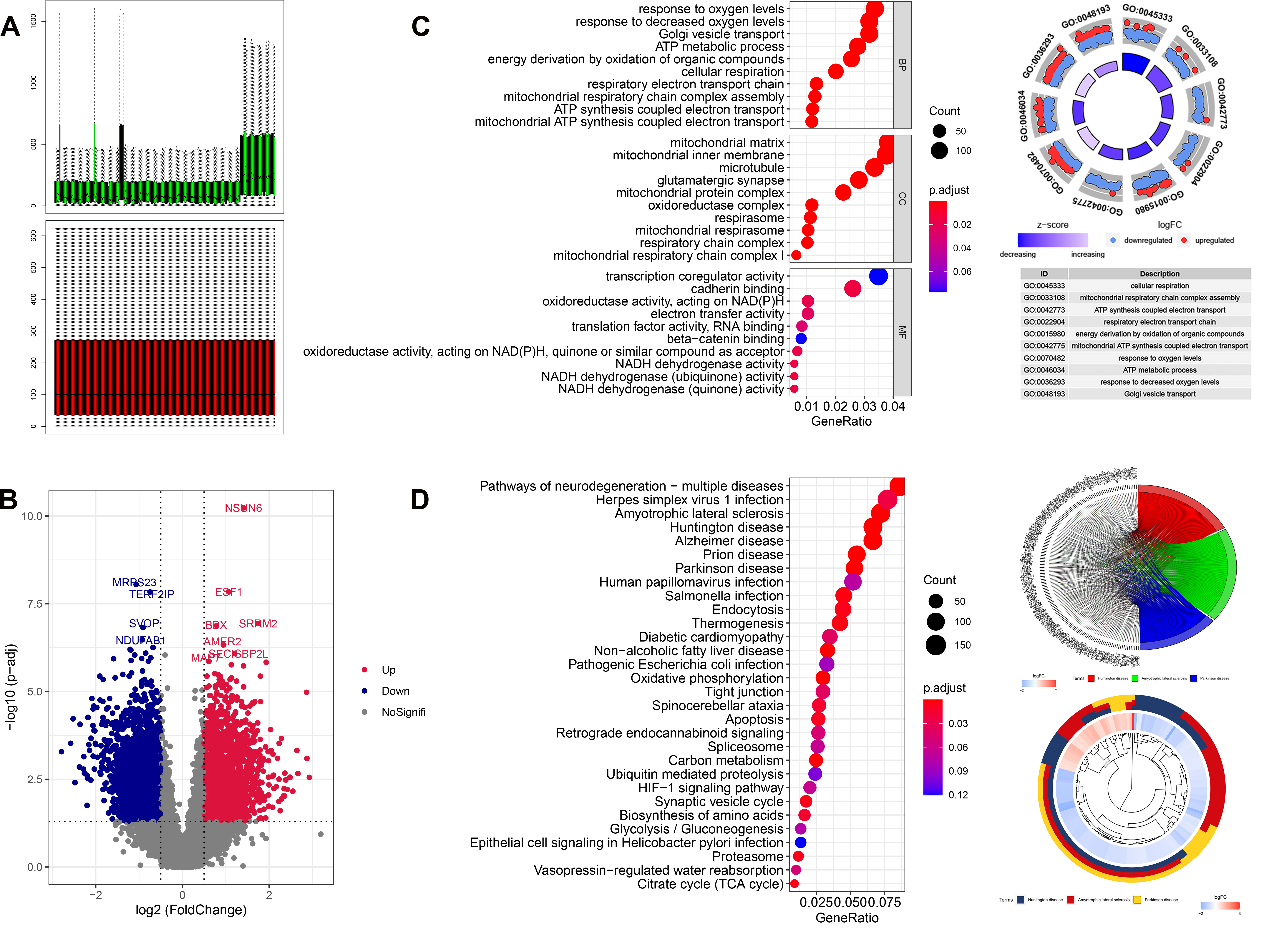


Figure S1. The DEGs and pathways in AD. A, B, Volcano plot of gene expression profile data between AD and normal samples. C, GO enrichment analysis. D, KEGG enrichment analysis.


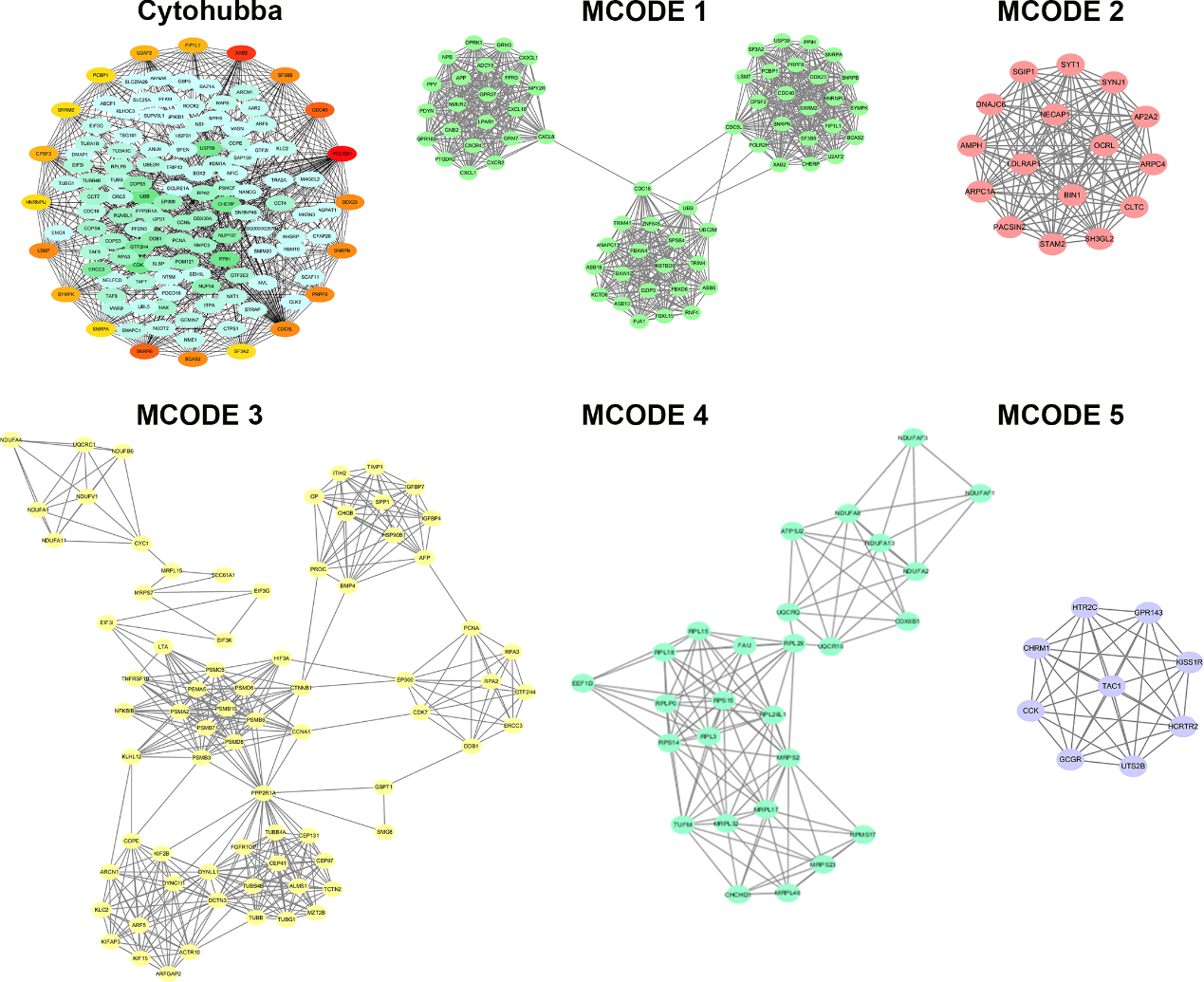


Figure S2. The hub gene network and the top 5 MCODE in AD.


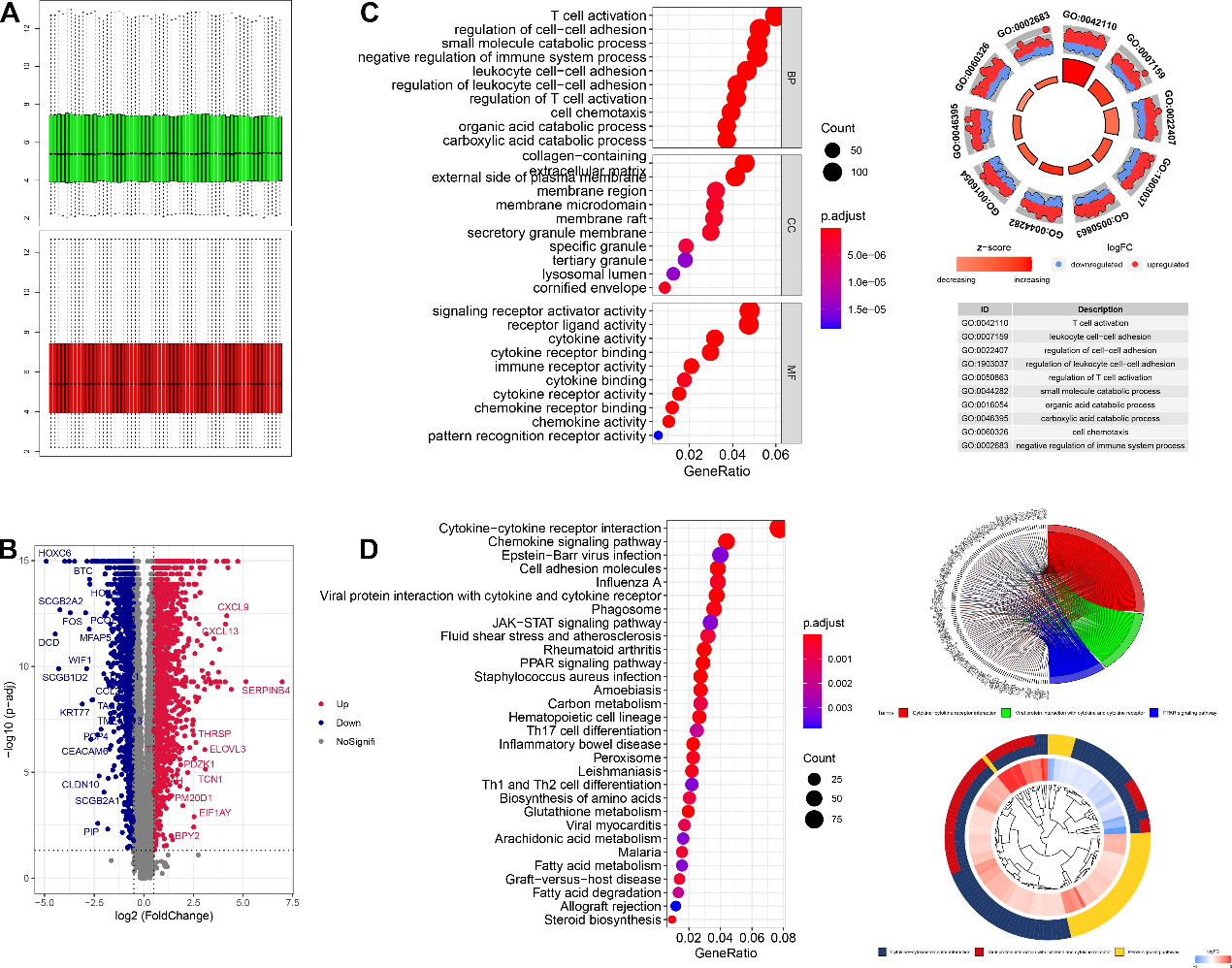
Figure S3. The DEGs and related pathways in rosacea. A, B, Volcano plot of gene expression profile data between rosacea and control samples. C, GO enrichment analysis. D, KEGG enrichment analysis.


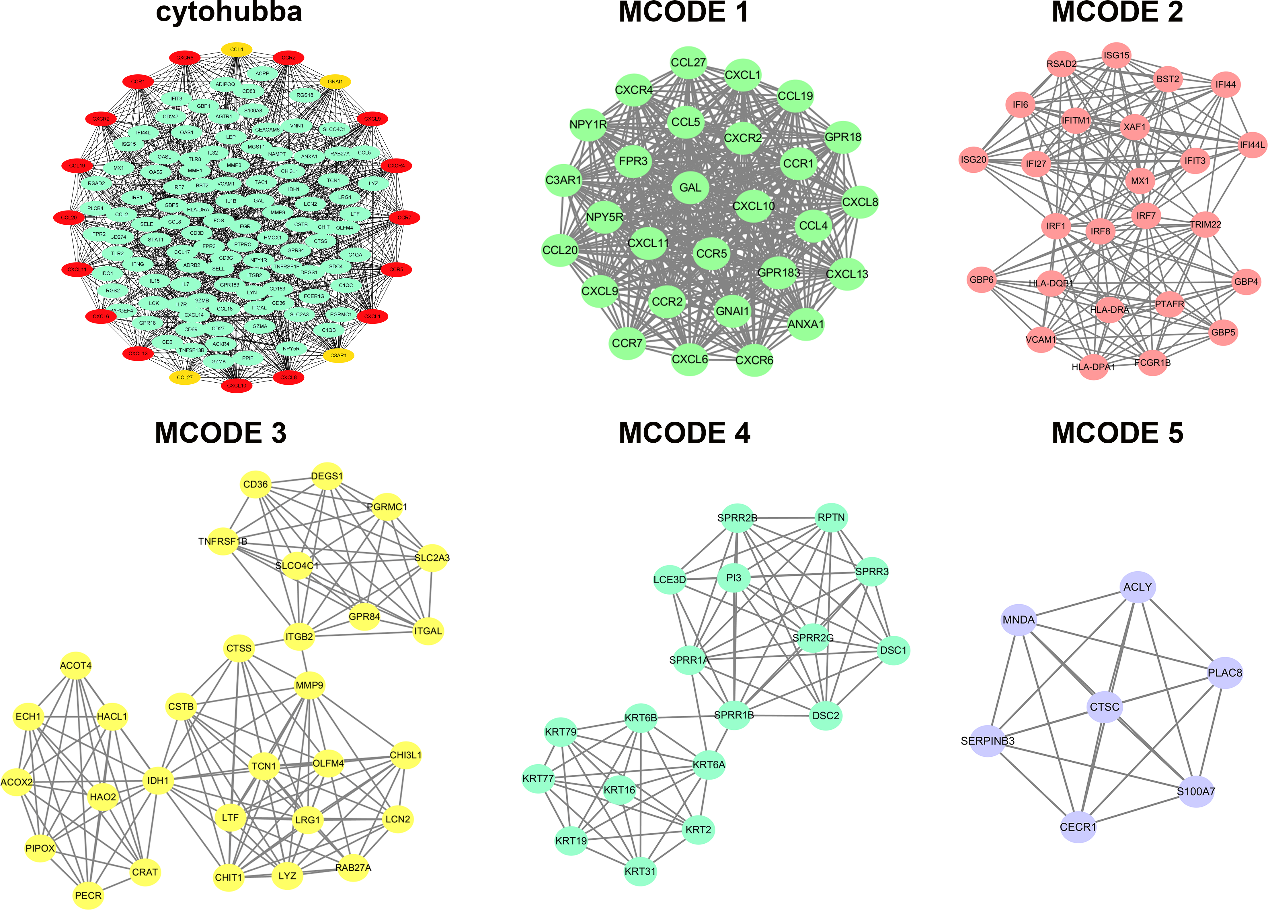


Figure S4. The hub gene network and the top 5 MCODE in rosacea.


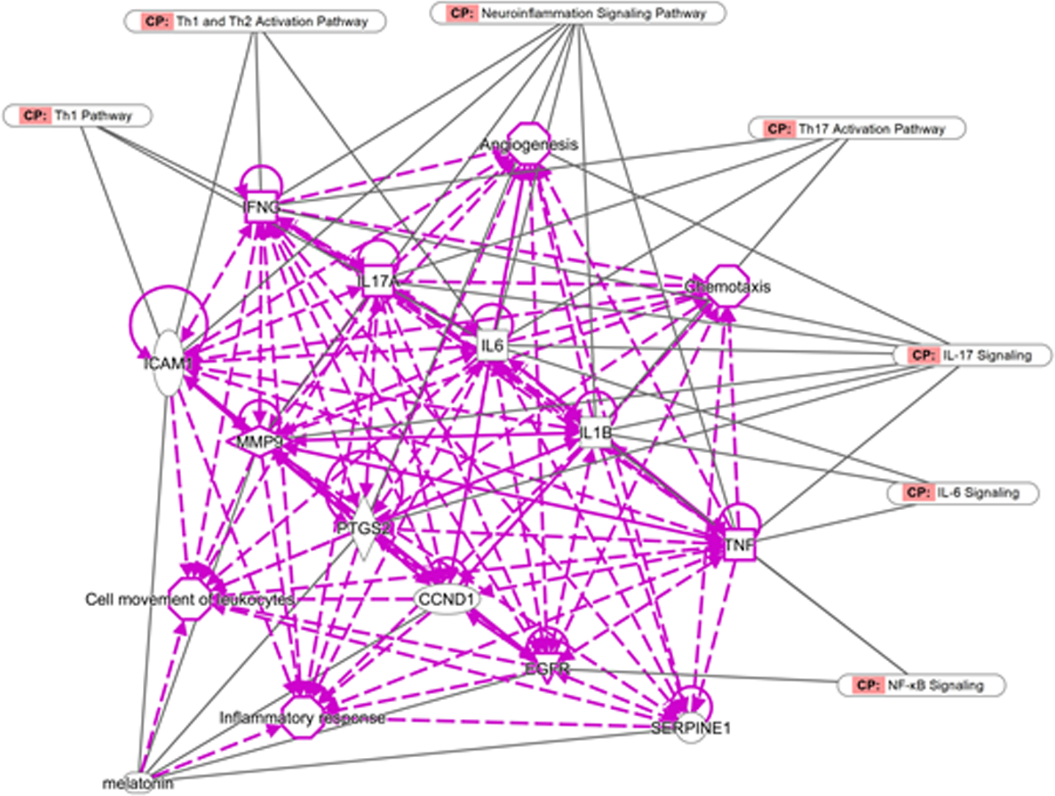


Figure S5. MLT-regulated pharmacological network in AD and rosacea
